# Ethnic variations in duration of untreated psychosis: report from the CRIS-FEP study

**DOI:** 10.1007/s00127-020-01922-9

**Published:** 2020-07-17

**Authors:** Sherifat Oduola, Tom K. J. Craig, Craig Morgan

**Affiliations:** 1 School of Health Sciences, University of East Anglia, Norwich Research Park, Norwich, NR4 7TJ UK; 2 Department of Health Service and Population Research, Institute of Psychiatry, Psychology and Neuroscience, King’s College London, De Crespigny Park, Denmark Hill, London, SE5 8AF UK; 3 South London and Maudsley NHS Foundation Trust, Biomedical Research Centre, Mapother House, De Crespigny Park, Denmark Hill, London, SE5 8AF UK

**Keywords:** Psychosis, Social determinants, Duration of untreated psychosis, Ethnicity, Pathways to care

## Abstract

**Objectives:**

There is inconsistent evidence on the influence of ethnicity on duration of untreated psychosis (DUP). We investigated ethnic differences in DUP in a large epidemiological dataset of first episode psychosis patients in an inner city area of south London, UK.

**Methods:**

We analysed data on 558 first episode psychosis patients at the South London and Maudsley NHS Trust, between 2010 and 2012. We performed multivariable logistic regression to estimate the odds of a short DUP (≤ 6 months) by ethnic group, controlling for confounders.

**Results:**

There was no evidence that ethnicity is associated with duration of untreated psychosis. However, we found evidence that a short DUP was strongly associated with age, living circumstances, and pathways to care variables (involuntary admission, out of office hour contact, accident and emergency referral, criminal justice agency referral and family involvement in help-seeking). Conversely, a long DUP was associated with report of social isolation, living alone, being single and General Practitioner referral.

**Conclusion:**

Our findings suggest that indicators of social isolation were associated with long DUP. Our data also show that pathways into care characteristics play significant role in DUP. Thus, the challenge of tackling the issue of timely access to EI under the new Access and Waiting Time standard for psychosis requires a multilevel approach, including joint working with communities, public awareness of psychosis, less restrictive referral pathways and adequate resourcing of early intervention for psychosis services. These will go a long way in addressing patients’ needs rather than be determined by service structures.

## Introduction

Duration of untreated psychosis (DUP) is a major contributor to the variation in outcomes following first episode psychosis [1, 2] and a robust predictor of poor outcomes [3, 4]. Therefore, reducing DUP has become an international priority and much effort has been spent assessing the impact of early intervention services on DUP. As such, in the last decade, a number of studies have been carried out evaluating patient-level [5, 6] and service-level [1, 7] factors associated with DUP and the effectiveness of early intervention (EI) service for psychosis in reducing DUP.

The central tenet of early intervention for psychosis is that it will improve the short- and long-term outcome of psychosis by (a) early detection and reduction in delays to receiving treatment (including DUP) and (b) optimising medical and psychosocial phase-specific treatments, modified as necessary specifically for use with people at an early stage in the illness [8, 9]. Over the last few decades, we have seen the establishment of early intervention programmes for psychosis, particularly in western countries, several of which have been carefully evaluated [6, 10–13]. These evaluations show relatively consistent findings of improved clinical and functional outcomes with reduced hospital admissions, relapse rates, symptom severity, and improved access to care [14]. But perhaps surprisingly, very few of these studies have explored variation in service access or outcome by ethnicity. A notable exception is the Lambeth Early Onset (LEO) study [6]. The LEO study showed that those in the EI group were less likely to drop out of care than patients receiving standard care, this difference was almost entirely accounted for by people of black African and Caribbean backgrounds.

Whilst there has been a stream of research evidence suggesting that, compared to the majority population, people from minority ethnic backgrounds are more likely to come in contact with mental health services via emergency routes such as emergency department and police referral [15–18], and less likely to have general practitioner (GP) involvement in their referral to specialist mental healthcare [16], only a few studies have reported on associations between DUP and ethnicity [19–23]. The findings are heterogeneous, with some reporting no difference in DUP by ethnicity [22, 23], some reported shorter DUP among black African and Asian patients [19, 21, 24], and others reported longer DUP among black Caribbean patients [25] and white British patients [5, 21, 24]. A number of explanations have been considered for the prolonged DUP and treatment delay among white British and black Caribbean patients, including stigma, the tendency to seek help via GP, living with family which may help reduce burden of the illness and help manage symptoms [5, 25, 26]. However, a handful of studies have shown the role of family stigma in some ethnic groups on help-seeking delays for psychosis, and potentially leading to poor clinical and social functioning outcomes [27, 28]. It has also been suggested that black African patients tend to experience acute onset of psychosis leading to more rapid presentation to services, which may explain the shorter DUP [21, 29]. However, given the known variations of sociodemographic and pathways to care characteristics across ethnic groups, it is crucial to consider the potential confounding effects of these variables in DUP by ethnic group. Of the available primary research on ethnicity and DUP, only two studies in the UK [19, 21] have disaggregated ethnic groups and taken into account the potential effects sociodemographic [19, 21] and pathways to care [21] factors on DUP. From these two studies, the effects of ethnicity on DUP varied, as outlined earlier. However, the reported tendency of acute onset of psychotic illness among the black African and Caribbean patients to explain the shorter DUP in these groups requires further research, given the limited number of studies specifically investigating ethnic differences in DUP. Therefore, in this study, we used data from the Clinical Record Interactive Search—First Episode Psychosis (CRIS-FEP) study [30], a large epidemiological dataset of first episode psychosis patients in an inner city area of south London, UK, to clarify these issues, owing to the potential service and policy implications.

## Methods

### Samples

The study sample was drawn from two inner city areas of southeast London, UK, namely the London boroughs of Lambeth (total population 303,086) and Southwark (total population 288,283) (ONS 2011a), served by the South London and Maudsley NHS Foundation Trust (SLaM).

### Study design, setting and participants

We employed a case register incidence study of patients with a first episode of any psychotic disorder (i.e. ICD F20-29, F30-33). The methods used in our case identification have been extensively described and published elsewhere [30–32]. In summary, we identified all patients presenting to the South London and Maudsley NHS Trust adult mental health services in Lambeth and Southwark for the first time with a psychotic disorder between May 2010 and April 2012 using the South London and Maudsley NHS Trust (SLaM) Clinical Records Interactive Search (CRIS) system [33], which provides fully anonymised access to all SLaM electronic clinical records.

### Inclusion and exclusion criteria

Our inclusion criteria were (a) resident in the London boroughs of Lambeth or Southwark, (b) aged 18–64 years (inclusive), (c) any psychotic disorder (i.e. ICD F20-29, F30-33) and (d) first contact with mental health services for psychosis. Exclusion criteria were (a) evidence of psychotic symptoms with an organic cause, (b) transient psychotic symptoms resulting from acute intoxication, and (c) previous contact with services for psychotic symptoms.

### Data

Data were collected by experienced researchers who were trained in the use of the data instruments for the purpose of data collection from clinical records. Data on demographic and social circumstances were collected using the Medical Research Council Socio-demographic schedule MRC-SDS [34]. Data relating to pathways to care encounters, duration of untreated psychosis, and specialist mental health service encounters were collected using the Personal and Psychiatric History Schedule (PPHS) [35].

The primary outcome in this study (i.e. duration of untreated psychosis) was calculated as the time from the onset of psychotic symptoms to first contact with any adult mental health service. For the purpose of analysis, first we summarised DUP as median and interquartile range in the whole sample and by study variables. Second, we dichotomised DUP as short (≤ 6 months) or long (> 6 months) and estimated the odds ratios by ethnic group. This was based on the evidence that the critical length of DUP that influences first episode psychosis outcome is 6 months [3].

Pathways to care characteristics were defined as follows: source of referral (i.e. GP, A&E, police/criminal justice agency), mode of contact with mental health service (i.e. inpatient vs. community), time of contact, involuntary admission and family involvement in help-seeking.

Ethnicity was coded using the MRC-SDC [34] according to the 18 categories used in the UK 2011 census. For analytical purposes, we collapsed the ethnic groups into seven as follows: white British, black Caribbean (black Caribbean and other black), black African, Asian (Indian, Pakistani, Bangladeshi, Chinese), white non-British (white Irish, white Gypsy, white Other), Other (Arab, Any Other Ethnic group), and Mixed (all mixed ethnic groups).

### Statistical analysis

Data were analysed using Stata version 15 [36]. Descriptive statistics and regression analyses were used. First, Chi-squared, *t*, and Kruskal–Wallis tests were used as appropriate to compare associations between DUP and key study variables (i.e. sociodemographic and pathways to care variables). Second, we used logistic regression to test the associations between duration of untreated psychosis and ethnicity, with complete data, adjusted for our a priori confounders (age, gender, employment status and living circumstances). Then we adjusted for pathways to care variables that were associated with DUP.

### Ethical approval

The CRIS system was approved as an anonymised dataset for secondary analysis by the Oxfordshire Research Ethics Committee (reference 08/H0606/71). Local approval for this study was obtained from the CRIS Oversight Committee at the BRC South London and Maudsley NHS Foundation Trust (reference: 09-041).

## Results

In total, 558 patients were identified as incident cases. There were more men [*n* 292 (52.3%)] than women, the largest proportions of the sample were black African patients [*n* 147 (26.3%)], unemployed (63.5%), and lived with family or friends (64.7%), and the overall mean age was 33.2 (sd 5.0) years. The median duration of untreated psychosis was 93 (IQR 19–447) days, 217 (38.9%) patients were referred to mental health services via the accident and emergency service and a similar proportion had an insidious onset of psychosis, 209 (37.5%). DUP as a binary outcome showed, *n* 338 (60.6%) experienced a short DUP (≤ 6 months).

### Associations between duration of untreated psychosis and study variables

Table 1 shows the associations between duration of untreated psychosis and the study variables. Kruskal–Wallis tests showed strong evidence of association between DUP and sociodemographic and pathways to care factors. Notably, the median DUP was longer for unemployed patients, those living alone, patients with a report of social isolation and those referred by GP. Conversely, patients with shorter DUP were more likely to be younger, students, employed and living with others. A shorter DUP was also associated with involuntary admission, out of office hours contact (i.e. presentations to services outside of normal working office hours), family involvement, A&E referral, criminal justice system referral and inpatient contact. There was no evidence of differences in DUP by ethnic group or EI service use status.

**Table 1:** Associations between duration of untreated psychosis, sociodemographic and pathways to care characteristics

Characteristics	*N* = 558 (%)	Median (IQR) days	Kruskal–Wallis test	*df*	*p*
Gender					
Men	292 (52.3)	106 (21–537)	2.35	1	0.12
Women	266 (47.7)	88 (17–354)			
Ethnicity					
White British	133 (23.8)	105 (22–514)	2.40	6	0.87
Black African	147 (26.3)	88 (17–447)			
Black Caribbean	91 (16.3)	126 (28–449)			
White non-British	75 (13.4)	86 (14–408)			
Asian	44 (7.9)	76.5 (8.5–243.5)			
Mixed	27 (4.8)	92 (23–361)			
Other	41 (7.3)	60 (12–560)			
Relationship status^1^					
Single	331 (62.2)	101 (21–410)	5.46	2	0.06*
Married/steady relationship	127 (23.8)	59 (8–362)			
Divorced/separated	74 (13.9)	119.5 (26–608)			
Employment^2^					
Unemployed	346 (68.5)	119 (28–492)	13.98	2	< 0.001
Student	60 (11.9)	24.5 (6 -351.5)			
Employed	99 (19.6)	45 (6–426)			
Lives with^3^					
Alone	161 (29.7)	116 (30–531)	6.16	2	0.04**
Family/relatives	325 (60.1)	90 (14-370)			
Other	55 (10.2)	58 (10–515)			
Country of birth^4^					
UK born	243 (46.3)	112 (23–426)	1.78	1	0.18
Non-UK born	282 (53.7)	81.5 (14–472)			
Report of social isolation^5^					
Yes	248 (52.6)	132 (31–585)	18.25	1	< 0.001
No	223 (47.4)	61 (7–354)			
Time of FEP contact					
Office hours	363 (65.1)	133 (27–596)	29.96	1	< 0.001
Out of office hours	195 (34.9)	44 (6–229)			
Involuntary admission					
Yes	423 (75.8)	32 (4–190)	31.18	1	< 0.001
No	135 (24.2)	125 (23–539)			
GP					
Yes	195 (34.9)	188 (48–832)	36.79	1	< 0.001
No	363 (65.1)	58 (7–349)			
A&E					
Yes	220 (39.4)	36.5 (6–285.5)	31.44	1	< 0.001
No	338 (60.6)	133 (32–572)			
Police/criminal justice system					
Yes	73 (13.1)	58 (7–196)	5.63	1	0.01**
No	485 (86.9)	101 (21–492)			
Early intervention service use^6^					
No	334 (58.1)	92.5 (21–467)	0.72	1	0.39
Yes	222 (39.9)	91.5 (10–447)			

Missing data: ^1^26 patients, ^2^53 patients, ^3^17 patients, ^4^33 patients, ^5^87 patients, ^6^2 patients

*IQR* interquartile range, *df* degree of freedom, *p p* value

*p ≤ 0.1; **p ≤ 0.05; ***p ≤ 0.01

### Associations between ethnicity and study variables

We observed ethnic differences in sociodemographic characteristics by gender, age, employment status and country of birth. There were more Asian (56.8%) and black Caribbean (57.1%) women compared with white British women (48.9%). Patients of ‘mixed’ ethnic group were younger (mean age = 29.7; sd = 9.6) compared with white British patients (mean = 34.5; sd = 12.5). Unemployment was highest among black Caribbean (80.4%) and ‘other’ ethnic group patients (85.3%), compared with white British patients (63.9%). Minority ethnic group patients, i.e. Black African patients (83.8%), Asian patient (73.2%), white non-British patients (90.7%) and ‘other’ ethnic group patients (81.1%) were more likely to be non-UK born, compared with their white British counterparts (3.2%) (Table 2).

**Table 2:** Sociodemographic characteristics by ethnic group

	White British *n* = 133 (%)	Black African *n* = 147 (%)	Black Caribbean *n* = 91 (%)	White non-British *n* = 75 (%)	Asian *n* = 44 (%)	Mixed *n* = 27 (%)	Other *n* = 41 (%)	Chi-sq test	*df*	*p*
Mean age at FEP in years (SD)	34.5 (12.5)	32.4 (9.9)	33.4 (10.6)	34.9 (9.6)	32.4 (10.3)	29.7 (9.6)	31.9 (9.1)	12.56	6	0.05**
Gender										
Men	68 (51.1)	79 (53.7)	39 (42.9)	41 (54.7)	19 (43.2)	15 (55.6)	31 (75.6)	14.12	6	0.02**
Women	65 (48.9)	68 (46.3)	52 (57.1)	34 (45.3)	25 (56.8)	12 (44.4)	10 (24.4)			
Relationship status										
Single	84 (66.1)	82 (58.2)	54 (62.8)	45 (60.8)	23 (56.1)	20 (76.9)	23 (62.2)	11.71	12	0.46
Married/steady relationship	32 (25.2)	32 (22.7)	19 (22.1)	20 (27.0)	13 (31.7)	4 (15.4)	7 (18.9)			
Divorced/separated	11 (8.7)	27 (19.1)	13 (12.1)	9 (12.2)	5 (12.2)	2 (7.7)	7 (18.9)			
Employment										
Unemployed	78 (63.9)	84 (65.1)	66 (80.4)	47 (65.3)	24 (61.5)	18 (66.7)	29 (85.3)	20.05	12	0.06*
Student	15 (12.3)	19 (14.7)	8 (9.8)	5 (6.9)	8 (20.5)	3 (11.1)	2 (5.9)			
Employed	29 (23.8)	26 (20.2)	8 (9.8)	20 (27.8)	7 (18.0)	6 (22.2)	3 (8.8)			
University	31 (32.3)	28 (27.1)	9 (14.7)	18 (33.9)	11 (34.4)	5 (23.8)	6 (21.4)			
Lives with										
Alone	41 (32.0)	39 (27.1)	31 (35.2)	21 (28.8)	12 (27.3)	7 (26.9)	10 (26.3)	6.60	12	0.88
Family/relatives	78 (61.0)	88 (61.1)	49 (55.7)	42 (57.5)	27 (61.4)	18 (69.2)	23 (60.5)			
Other	9 (7.0)	17 (11.8)	8 (9.1)	10 (13.7)	5 (11.4)	1 (3.9)	5 (13.2)			
Country of birth										
UK born	122 (96.8)	23 (16.2)	52 (64.2)	7 (9.3)	11 (26.8)	21 (91.3)	7 (18.9)	268	6	< 0.001
Non-UK born	4 (3.2)	119 (83.8)	29 (35.8)	68 (90.7)	30 (73.2)	2 (8.7)	30 (81.1)			
Report of social isolation										
Yes	49 (44.9)	66 (53.2)	44 (59.5)	32 (47.8)	22 (55.0)	14 (60.9)	21 (61.8)	6.46	6	0.37
No	60 (55.1)	58 (46.8)	30 (40.5)	35 (52.2)	18 (45.0)	9 (39.1)	13 (38.2)			

*p ≤ 0.1; **p ≤ 0.05; ***p ≤ 0.01

Table 3 compares pathways to care variables by ethnic group. Notably, we found that black African and ‘mixed’ patients were more likely to be admitted involuntarily, compared with their white British counterparts (34%, 33.3% and 17.3%, respectively). We found no ethnic differences in duration of untreated psychosis. In addition, there were no ethnic differences in mode of onset, source of referral, early intervention service use, family involvement in help-seeking or time of contact with mental health services.

**Table 3:** Pathways to care characteristics by ethnic group

	White British *n* = 133 (%)	Black African *n* = 147 (%)	Black Caribbean *n* = 91 (%)	White non-British *n* = 75 (%)	Asian *n* = 44 (%)	Mixed *n* = 27 (%)	Other *n* = 41 (%)	Chi-sq test	*df*	*p*
Median DUP in days (IQR)	105 (22–514)	88 (17–447)	126 (28–449)	86 (14–408)	76.5 (8.5–243)	92 (23–361)	60 (12–560)	2.40	6	0.87
Involuntary admission										
Yes	23 (17.3)	50 (34.0)	19 (20.9)	14 (18.7)	13 (29.9)	9 (33.3)	7 (17.1)	16.02	6	0.01**
No	110 (82.7)	97 (66.0)	72 (79.1)	61 (81.3)	31 (70.4)	18 (66.7)	34 (82.9)			
Time of contact										
Office hours	88 (66.2)	86 (58.5)	62 (68.1)	50 (66.7)	27 (61.4)	19 (70.4)	31 (75.6)	5.92	6	0.43
Out of hours	45 (33.8)	61 (41.5)	29 (31.9)	25 (33.3)	17 (38.6)	8 (29.6)	10 (24.4)			
Early intervention service use										
No	78 (85.6)	77 (52.4)	55 (61.1)	51 (68.0)	29 (67.4)	15 (55.6)	29 (70.7)	8.88	6	0.18
Yes	55 (41.4)	70 (47.6)	35 (38.9)	24 (32.0)	14 (32.6)	12 (44.4)	12 (29.3)			
Family involvement in help seeking										
Yes	44 (33.1)	48 (32.7)	39 (42.9)	29 (38.7)	17 (38.6)	8 (29.6)	11 (26.8)	5.27	6	0.50
No	89 (66.9)	99 (67.4)	52 (57.1)	46 (61.3)	27 (61.4)	19 (70.4)	30 (73.2)			
GP referral										
No	83 (62.4)	102 (69.4)	60 (65.9)	50 (66.7)	29 (65.9)	17 (63.0)	21 (51.2)	5.23	6	0.51
Yes	50 (37.6)	45 (30.6)	31 (34.1)	25 (33.3)	15 (34.1)	10 (37.0)	20 (48.8)			
A&E referral										
No	81 (60.9)	85 (57.8)	61 (67.0)	45 (60.0)	23 (52.3)	18 (66.7)	28 (68.3)	4.73	6	0.57
Yes	52 (39.1)	62 (42.2)	30 (33.0)	30 (40.0)	21 (47.7)	9 (33.3)	13 (31.7)			
Police/criminal justice system referral										
No	117 (88.0)	123 (83.7)	80 (87.9)	64 (85.3)	39 (88.6)	21 (77.8)	37 (90.2)	3.80	6	0.72
Yes	16 (12.0)	24 (16.3)	11 (12.1)	11 (14.7)	5 (11.4)	6 (22.2)	4 (9.8)			
Other referral										
No	118 (88.7)	131 (89.1)	72 (79.1)	66 (88.0)	41 (93.2)	25 (92.6)	37 (90.2)	8.75	6	0.27
Yes	15 (11.3)	16 (10.9)	19 (20.9)	9 (12.0)	3 (6.8)	2 (7.4)	4 (9.8)			
Mode of contact										
Community	84 (63.2)	78 (53.1)	60 (65.9)	39 (52.0)	23 (52.2)	13 (48.1)	28 (68.3)	9.83	6	0.13
Inpatient	49 (36.8)	69 (46.9)	31 (34.1)	36 (48.0)	21 (47.7)	14 (51.8)	13 (31.7)			
Mode of onset										
Acute (within a week)	25 (18.8)	33 (22.6)	18 (19.8)	15 (20.0)	11 (25.0)	4 (14.8)	10 (24.4)	11.68	18	0.86
Moderate (within a month)	26 (19.6)	29 (19.8)	16 (17.6)	18 (24.0)	7 (15.9)	6 (22.2)	9 (21.9)			
Gradual (up to 6 months)	25 (18.8)	36 (24.5)	22 (24.2)	13 (17.3)	12 (27.3)	9 (33.3)	5 (12.2)			
Insidious (more than 6 months)	57 (42.8)	49 (33.3)	35 (38.4)	29 (38.7)	14 (31.8)	8 (29.6)	17 (41.5)			

*p ≤ 0.1; **p ≤ 0.05; ***p ≤ 0.01

### Associations between duration of untreated psychosis and ethnicity

First, we estimated the unadjusted and adjusted odds ratios for long vs. short DUP for whom data were complete (Table 4). Here, we observed that Asian patients experienced a short DUP (Model 1, Table 4). However, this attenuated somewhat when we adjusted for sociodemographic factors (Model 2, Table 4). The evidence no longer holds when we adjusted for pathways to care variables (Model 3, Table 4). There was no further evidence of ethnic differences in DUP. Given that we observed strong associations between key social indicators (e.g. unemployment and living alone) and DUP and ethnicity. We examined interactions between ethnicity and living circumstance, as well as ethnicity and employment status and fitted an interaction terms as follows: (ethnicity × living circumstances) and (ethnicity × employment status). We did not find evidence of effect modifications between the main effects and interaction terms for ethnicity and DUP (LR test *X*^2 ^ =  7.10, *p* =  0.31) and (LR test *X*^2^ =  3.27, *p* =  0.77), respectively.

**Table 4:** Unadjusted and adjusted odds ratios of associations between ethnicity and duration of untreated psychosis (*n*** = **492)

Ethnicity	OR (95% CI); model 1	Adj. OR (95% CI); model 2	Adj. OR (95% CI); model 3
White British	1.00	1.00	1.00
Black African	0.7 (0.4–1.2)	0.8 (0.4–1.3)	0.8 (0.5–1.4)
Black Caribbean	0.9 (0.5–1.6)	0.9 (0.5–1.7)	1.0 (0.6–1.9)
White non-British	0.7 (0.4–1.3)	0.7 (0.3–1.3)	0.8 (0.4–1.5)
Asian	0.4 (0.2–1.0)*	0.5 (0.2–1.1)	0.5 (0.2–1.3)
Mixed	0.6 (0.2–1.5)	0.6 (0.2–1.6)	0.6 (0.2–1.7)
Other	0.8 (0.3–1.8)	0.7 (0.3–1.7)	0.6 (0.3–1.4)

Model 1—unadjusted

Model 2—adjusted for age, gender, employment status and living circumstances

Model 3—adjusted for age, gender, employment status, living circumstances, family involvement, GP referral, A&E referral, mode of contact and time of contact

*CI* confidence intervals, *OR* odds ratio, *Adj. OR* adjusted odds ratio

*p ≤ 0.1; **p ≤ 0.05; ***p ≤ 0.01

## Discussion

### Main findings

In this study, our results suggest that there were no ethnic differences in DUP. There was no evidence of ethnic differences in the mode of onset of psychosis. However, there were striking differences in DUP according to other sociodemographic and pathways to care characteristics, a greater proportion of patients with long DUP had encountered mental health services via their GPs, were unemployed, living alone and had a report of social isolation. Conversely, contacts with mental health services for psychosis via emergency and crisis services (e.g. A&E, criminal justice system, involuntary admission) and family involvement were associated with shorter DUP.

### Methodological considerations

Several studies have investigated ethnic differences in DUP and pathways to care, but they have either focussed on hospital admission or EI only samples. Our study is the most comprehensive to date, using electronic health records to identify all persons presenting with psychosis to a large mental health service provider to an inner city population of 1.3 million people. The findings are strengthened by the large sample size that enabled analyses of ethnicity according to the UK  2011 Census, which afforded the investigation of pathways to care by specific ethnic groups rather than broadly defined categories, particularly for the black African and black Caribbean groups.

Our findings should be interpreted with a few limitations in mind. A key methodological consideration is the measurement of DUP. Whilst we have carefully dated the start and end point of DUP, using a standardised instrument, there may still be measurement bias, particularly if the clinical information varies by ethnic group. The cross-sectional nature of our data collection is another limitation of the study. While we adjusted for sociodemographic and pathways to care factors, our results could still be confounded by unmeasured factors such as previous service use for non-psychotic disorder. Further, our findings among the Asian patients may not be generalizable owing to the heterogeneity in our Asian group. We included Chinese people as well as Indian, Pakistani and Bangladeshi people. Another limitation is in our data source, while CRIS provides comprehensive information on service utilisation, it is noteworthy that the clinical data are recorded by clinicians for clinical purposes and not collected for research; therefore, the accuracy of information depends on the quality of clinicians’ documentation.

### Explaining the findings

#### DUP and ethnicity

Few studies have found relationships between DUP and ethnicity [19, 21, 24, 37]. Our findings of shorter DUP among Asian patients are consistent with the findings by Ghali et al. [21] who, in a sample of 775 early intervention patients, also found a shorter median DUP among Asian patients (60 days) compared with white British patients (113 days).

Our findings of no difference in DUP among black African and black Caribbean patients are in keeping with a recent Canadian study [22]. We also found no difference in DUP among other minority ethnic group patients, which has been echoed in other studies [3, 20, 23]. However, in contrast to the findings by Morgan and colleagues in Aetiology and Ethnicity in schizophrenia and Other Psychosis (AESOP) study, carried out nearly two decades ago in the same catchment area as our study [19], we did not find evidence of a short DUP among black African patients. Similarly, in an East London study, Bhui et al. [37] reported shorter DUP among the broadly defined black ethnic group patients, compared with white British patients. The lack of differences in DUP by ethnic group may be explained in a few ways. First, as our data show, except for involuntary admission, there were no ethnic differences in pathways to care in our sample, suggesting possible change over time in the catchment area. For example, earlier studies have shown trends towards the increased odds of black African patients having police and the criminal justice system involved in their pathways to care [15, 21, 38]. Several of these studies were carried out before or at the early stages of the introduction of early intervention for psychosis services in the UK. EI services act to detect and reduce delays to receiving treatment for people at an early stage in the psychotic illness [1, 6]. As part of their outreach work, EI services are also known to work collaboratively with other agencies such as the criminal justice system and emergency rooms to identify people at the early phase of psychosis [39]. Whilst, a short DUP was strongly associated with urgent/crisis pathways to care in our data, it is not surprising that EI engagement at population and service level may be reducing ethnic disparities in DUP. Second, indicators of social isolation play significant role in DUP and how people contact mental health services, regardless of ethnicity, as we discuss later. However, it is noteworthy to consider cultural and illness beliefs of psychosis in some ethnic groups. For example, in some societies (e.g. African, South America, Caribbean), it is reported that people believe that mental illness could be caused either by spirits or supernatural powers [40–42]; hence, such beliefs will inevitably influence help-seeking behaviour [43]. Third, the variations in the definitions of DUP in our study and some previous studies may also play a part in the lack of ethnic differences in DUP, and this issue was highlighted in a recent systematic review of studies of ethnic differences in DUP [44].

#### DUP, pathways to care and indicators of social isolation

Our findings show that people who were referred to mental health services by the GP had longer DUP, but those seeking help via A&E, criminal justice system, involuntarily admitted and had family involvement in help-seeking experienced a shorter DUP. There are a number of possible explanations for this observation. First, people who are working or studying may feel that the local A&E is more accessible rather than wait for a GP appointment, consequently resulting in higher consultations in A&E for all conditions [45]. It also possibly reflects acuity of illness, such that those with abrupt onset and/or with socially disruptive behaviours are taken to A&E by alarmed family members or the criminal justice system, e.g. police. Second, the findings of help-seeking via A&E might also suggest that it is the most accessible service to recent migrants to the UK. This has also been reported in previous studies [21–23, 46]. Third, it is possible that living alone may be synonymous with social isolation and stigma; therefore help-seeking may be delayed by people in such situation. The impact of stigma as a barrier to help-seeking has been established at individual level [47, 48].This extends to stigma experienced at service level by people presenting acute general hospitals with mental health complaint [49]. The link between stigma, social isolation and psychosis is well established [50, 51]. However, our data also show that a sizeable proportion of people accessing care in crisis do so out of office hours and are accompanied by family or friends, these people also experienced shorter periods of DUP. This is not surprising, as it has long been clear that the involvement of significant others in help-seeking ameliorates negative pathways to care [52, 53]. The use of emergency services also highlights the lack of availability of and accessibility to specialist mental health services. In recent decades, the structure and provision of mental health services have changed significantly. For example, psychiatric emergency services [54], which provided walk-in clinics for people in need of urgent mental health assessment and support within the premises of psychiatric hospitals were replaced with crisis resolution and home treatment teams that largely accept patients by referrals only [55]. Meanwhile, accessibility to the crisis resolution teams have been criticised by patients and carers expressing that these services are not sufficiently accessible to them particularly out of normal working hours [56].

#### Implications for clinical services

The evidence of association between a long DUP and GP referral calls for an urgent improvement in access to timely treatment for psychosis. Not only campaigns targeted at primary care clinicians, but initiatives that employ a multifaceted approach in raising awareness of mental health in the population. For example, healthcare providers need to move beyond generic health education programmes but work closely with community groups to improve pathways to care for first episode psychosis patients. Such collaborative and population-level approach to integrating statutory healthcare services with community and third-sector groups will help address the influence of social factors (e.g. unemployment, isolation, living alone) on DUP, shown in our study, and consequently improve outcomes. There are opportunities to engage with community leaders and religious groups, who have been found to be significant in how people from some minority ethnic groups seek help for mental health distress [20].

We acknowledge that this study was conducted prior to the introduction of new Access and Waiting Time Standard for early intervention for psychosis services in England [57]. The Access and Waiting Time standard was introduced by the UK government in 2016, to extend the age of accepting people presenting with first episode psychosis to early intervention for psychosis services from 18 to 35 years to 18–64 years. Nonetheless, our data, which include people up to the age of 64 years, provide an insight into service provision gaps. While we did not find evidence of differences in DUP and EI service use status, we found that the majority of people with short DUP accessing mental health services came in via emergency service such as the A&E, police and criminal justice service and out of hour contact. This suggests that acute onset and presentation of psychosis may be a significant indicator for accessing specialist service. This is reflected in findings from recent studies, following the implementation of Access and Waiting Time standard, which suggest that patients aged > 35 years present with complex needs [58, 59]. However, further research is needed to shed light on which factors influence pathways to care for patients over 35 years.

Based on our analyses, the challenge of addressing the issue of timely access to early psychosis services EI will need a multilevel approach, including joint working with communities, public awareness of psychosis, less restrictive referral pathways and adequate resourcing of EI services.

## Conclusion

Our findings show there is no strong evidence that ethnicity is associated with duration of untreated psychosis. Longer DUP was associated with GP referral, which may reflect the ongoing pressures on resources and waiting times for consultation in primary care. As the gateway to secondary care, primary care services need adequate funding and staffing to achieve the government’s GP Forward View [60]. Our findings are also relevant to international policies on mental health. For example, a widespread of early psychosis services in the United States is ongoing therefore; deploying early psychosis service with a whole community approach (set out earlier) in mind will be effective in meeting the needs of those who need such services the most, as well as addressing ethnic disparities in psychosis outcomes [61]. A long duration of untreated psychosis is also a reflection of an onset that goes unnoticed because the person has limited social network. Unsurprisingly, we found that people accessing mental health services via A&E department and often accompanied by family or friends experienced shorter DUP. Initiatives and services other than A&E that can increase access to early intervention for psychosis 24 h a day are essential. In addition, triage systems within mental health services that afford rapid transfer for patients exhibiting onset of psychosis to early intervention services are also important. These will go a long way in addressing patients’ needs rather than be determined by service structures.
